# High-pressure-induced water penetration into 3-­isopropylmalate dehydrogenase

**DOI:** 10.1107/S0907444912001862

**Published:** 2012-02-14

**Authors:** Takayuki Nagae, Takashi Kawamura, Leonard M. G. Chavas, Ken Niwa, Masashi Hasegawa, Chiaki Kato, Nobuhisa Watanabe

**Affiliations:** a Department of Biotechnology, Graduate School of Engineering, Nagoya University, Japan; b Synchrotron Radiation Research Center, Nagoya University, Japan; c Structural Biology Research Center, Photon Factory, High Energy Research Organization (KEK), Japan; d Department of Materials Science and Engineering, Graduate School of Engineering, Nagoya University, Japan; e Institute of Biogeosciences, Japan Agency for Marine-Earth Science and Technology (JAMSTEC), Japan

**Keywords:** high-pressure protein crystallography, pressure denaturation, water penetration, 3-isopropylmalate dehydrogenase

## Abstract

Hydrostatic pressure induces structural changes in proteins, including denaturation, the mechanism of which has been attributed to water penetration into the protein interior. In this study, structures of 3-isopropylmalate dehydrogenase (IPMDH) from *Shewanella oneidensis* MR-1 were determined at about 2 Å resolution under pressures ranging from 0.1 to 650 MPa using a diamond anvil cell (DAC). Although most of the protein cavities are monotonically compressed as the pressure increases, the volume of one particular cavity at the dimer interface increases at pressures over 340 MPa. In parallel with this volume increase, water penetration into the cavity could be observed at pressures over 410 MPa. In addition, the generation of a new cleft on the molecular surface accompanied by water penetration could also be observed at pressures over 580 MPa. These water-penetration phenomena are considered to be initial steps in the pressure-denaturation process of IPMDH.

## Abstract 2

Structures of 3-­isopropylmalate dehydrogenase were determined at pressures ranging from 0.1 to 650 MPa. Comparison of these structures gives a detailed picture of the swelling of a cavity at the dimer interface and the generation of a new cleft on the molecular surface, which are accompanied by water penetration.

## 1. Introduction

A hydrostatic pressure of several hundred megapascals has been reported to induce structural changes in proteins such as conformational changes, dissociation of oligomeric proteins and denaturation (Boonyaratanakornkit *et al.*, 2002 ▶; Akasaka, 2006 ▶). On the basis of experimental studies and simulations (Frye & Royer, 1998 ▶; Paliwal *et al.*, 2004 ▶), these effects are currently considered to be mainly caused by the penetration of water molecules into the protein interior under high pressure. The solution structure of ubiquitin determined at 300 MPa by high-pressure NMR spectroscopy (Kitahara *et al.*, 2005 ▶) was marked by a conformational change from a closed to an open form of ubiquitin. Based on this high-pressure structure of ubiquitin, computational studies were performed using the three-dimensional reference interaction-site model (RISM) theory. The results of this investigation illustrated the penetration of water molecules into the hydrophobic interior of ubiquitin at high pressure, confirming an alteration of the hydration of proteins under high pressure (Imai *et al.*, 2007 ▶). Notably, these simulations demonstrated how water penetration reduces the partial molar volume (PMV) of ubiquitin.

In the past decades, high-pressure protein crystallography (HPPX) has been established and applied to structural studies of several macromolecules (Kundrot & Richards, 1986 ▶, 1987 ▶; Katrusiak & Dauter, 1996 ▶; Fourme *et al.*, 2001 ▶, 2009 ▶; Urayama *et al.*, 2002 ▶; Collins *et al.*, 2005 ▶, 2011 ▶; Girard *et al.*, 2007 ▶, 2010 ▶; Ascone *et al.*, 2010 ▶; Kurpiewska & Kewiński, 2010 ▶). It is believed that the HPPX method can be a powerful tool to elucidate the mechanism of the effects of high pressure on proteins owing to the fact that it allows direct observation of structured water molecules. An example is illustrated by the structure determination of T4 lysozyme carrying the L99A mutation-specific large hydrophobic cavity, for which water penetration was observed at 200 MPa (Collins *et al.*, 2005 ▶). In this structure, the volume of the cavity at 200 MPa was reduced by less than 3% compared with that in the structure at atmospheric pressure, while additional conformation changes of the protein itself were scarcely induced. Another example of HPPX measurements is represented by structure determination of the urate oxidase (UOX) tetramer at 150 MPa (Girard *et al.*, 2010 ▶). In contrast to L99A T4 lysozyme, water penetration into the hydrophobic cavity of UOX was not observed in the crystal structure at high pressure, although structure changes occurred in a polar pocket neighbouring the hydrophobic cavity, which expanded to 111% of its original volume even though the pressure was increased.

In addition to studies of the effects of pressure on proteins from land organisms, which live in an ambient environment, detailed analyses have also been performed on proteins from deep-sea organisms that have evolved at higher pressures. Interestingly, most enzymes originating from deep-sea organisms remain active at high pressure, while related enzymes from land organisms lose their activity (Kato *et al.*, 2008 ▶). For example, 3-isopropylmalate dehydrogenase (IPMDH) from the obligate piezophile *Shewanella benthica* DB21MT-2, originally isolated from the Mariana Trench (Kato *et al.*, 1998 ▶; Nogi & Kato, 1999 ▶), was shown to be more tolerant to high-pressure stress than the same enzyme originating from the nonpiezophile *S. oneidensis* MR-1 (Kasahara *et al.*, 2009 ▶). IPMDH catalyzes the reduction of 3-isopropylmalate (IPM) to 2-­isopropyl-3-oxosuccinate in the presence of divalent metal cations such as magnesium or manganese ion and nicotin­amide adenine dinucleotide. In order to elucidate the pressure tolerance of proteins from deep-sea organisms, we have initiated a series of structural studies on several IPMDHs using the HPPX method. In the present report, we attempt to underpin mechanisms explaining the regulation of pressure-induced effects on the IPMDH from the nonpiezophile *S. oneidensis* MR-1 (SoIPMDH).

## 2. Materials and methods

### 2.1. Purification and crystallization of SoIPMDH

SoIPMDH was overexpressed in *Escherichia coli* BL21-CodonPlus-(DE3)-RIL cells (Agilent Technologies, Santa Clara, California, USA) transformed with pQE-MR1-*leuB* vector. The pQE-MR1-*leuB* vector carries a subcloned *leuB* gene of *S. oneidensis* MR-1 inserted into the *Bam*HI/*Hin*dIII restriction sites of the pQE-80L vector (Qiagen, Hilden, Germany), which allows the expression of N-terminally His_6_-tagged fusion protein (Kasahara *et al.*, 2009 ▶).

The cells were grown at 310 K in LB medium containing 100 mg l^−1^ ampicillin. Expression of SoIPMDH was induced using 0.5 m*M* IPTG. The cells were cultivated for 6 h after induction and then harvested by centrifugation at 4000*g* for 15 min. To remove residual broth, the cell pellets were washed with cell-lysis buffer (50 m*M* Tris–HCl pH 8.0, 150 m*M* NaCl) and kept frozen until further purification.

The cells were thawed on ice, suspended in cell-lysis buffer supplemented with lysozyme (0.1 mg ml^−1^ final concentration) and incubated for 30 min before being lysed by sonication. Cell debris was removed by centrifugation (15 000*g*, 277 K, 30 min). The protein was then purified by Ni-affinity chromatography and gel-filtration chromatography.

Large crystals of the SoIPMDH–IPM complex were grown by the microseeding method. Seed crystals were obtained *via* the hanging-drop vapour-diffusion method by mixing purified protein solution (15 mg ml^−1^ in 10 m*M* magnesium chloride, 10 m*M* IPM, 10 m*M* Tris–HCl pH 8.0) with reservoir solution [11%(*w*/*v*) PEG 3350, 200 m*M* calcium acetate, 100 m*M* Na HEPES pH 7.0] in a 1:1 ratio. The crystals grew to typical dimensions of 0.15 × 0.15 × 0.03 mm within 4–5 d and belonged to space group *C*2, with unit-cell parameters *a* = 104.80, *b* = 59.00, *c* = 76.75 Å, β = 119.7°.

### 2.2. Data collection at atmospheric pressure

Prior to data collection, the crystal was soaked in a solution consisting of 18%(*w*/*v*) PEG 3350 in 100 m*M* Na HEPES pH 7.0, 200 m*M* calcium acetate, 10 m*M* magnesium chloride, 10 m*M* IPM and then sealed in a glass capillary together with a small amount of the soaking solution. Diffraction data were collected at room temperature using a Rigaku FR-E Cu *K*α X-­ray source equipped with a Rigaku R-­AXIS VII detector. Three crystals were used for data collection in order to record a complete data set with limited radiation damage.

### 2.3. Data collection at higher pressures

The high-pressure environment used in this study was generated by a diamond anvil cell (DAC) of the piston-cylinder type (Chervin *et al.*, 1995 ▶) with a culet diameter of 1 mm and a thickness of 1.49 mm (Boehler Almax type; Boehler & Hantsetters, 2004 ▶). The diameter and height of the sample chamber were about 0.6 and 0.3 mm, respectively. The resulting geometrical aperture of a cone with its vertex positioned at the centre of the sample chamber is about 90°.

The crystals were mounted in the DAC and slowly com­pressed. The solution used as a pressure medium consisted of 18%(*w*/*v*) PEG 3350 in 100 m*M* Na HEPES pH 7.0, 200 m*M* calcium acetate, 10 m*M* magnesium chloride, 10 m*M* IPM. The actual pressure in the sample chamber was determined using the wavelength shift of the fluorescence emanating from a ruby ball loaded into the chamber together with the protein crystals (Zha *et al.*, 2000 ▶). Pressure measurements were performed before and after X-ray measurements.

While performing HPPX measurements, several difficulties were noticed (Kurpiewska & Kewiński, 2010 ▶). (i) The pressure medium being liquid, crystals in the DAC were mobile and were moving during measurements of the X-ray diffraction. (ii) Recording highly complete data is difficult for crystals belonging to lower symmetry space groups such as *C*2 owing to the restricted aperture of the DAC, the angle of which is often less than 90°. (iii) Radiation damage during data collection is not negligible because the HPPX measurements are performed at room temperature.

**Figure 1:**
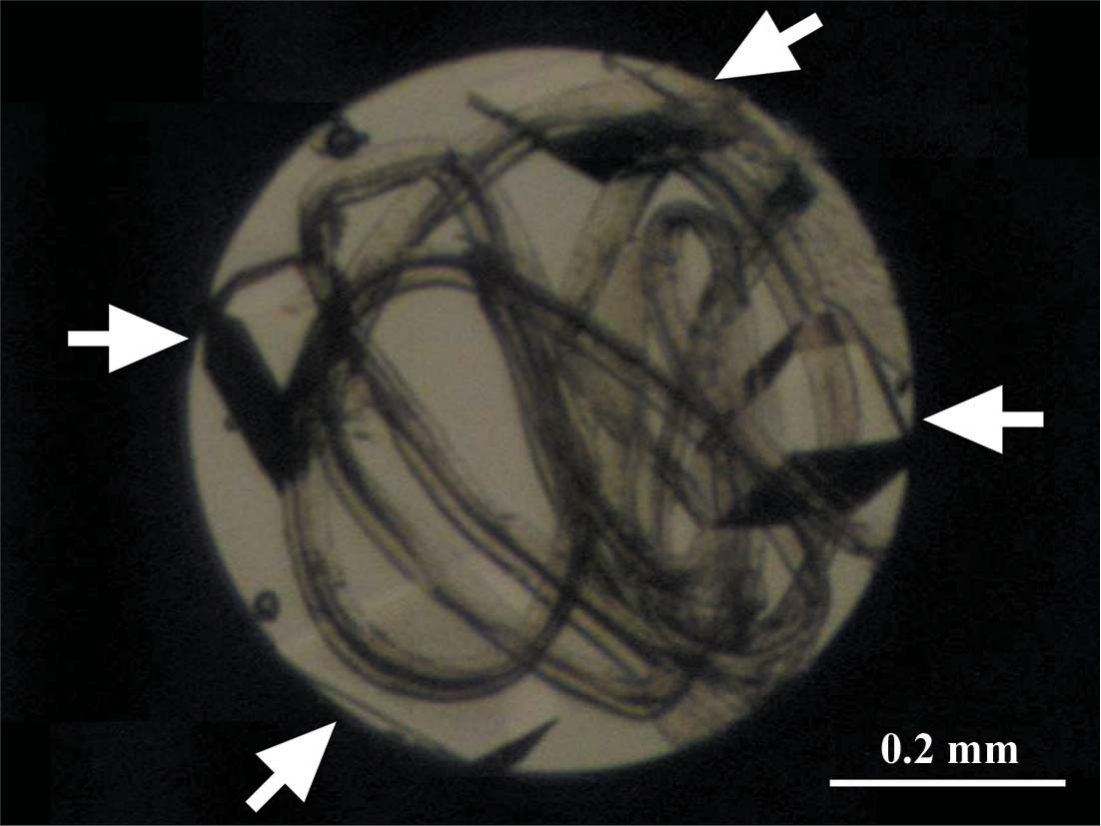
Photograph of the use of cigarette-filter fibres in the sample chamber. Four SoIPMDH–IPM crystals (indicated by arrows) are tiled with different orientations from each other and fixed in position using the knotted fibres.

HPPX experiments were performed on beamline AR-NW12A of the Photon Factory. Crystal movement in the DAC is significant at AR-NW12A because of the horizontal spindle axis of the goniometer. To prevent such motion, Katrusiak and Dauter used cotton wool fibres to fill in the chamber (Katrusiak & Dauter, 1996 ▶). As a substitute for cotton wool fibres, the crystals were placed into the sample chamber with a few cigarette-filter fibres, which had the advantage of being somewhat flexible and springy and could be tied into a loose knot (Fig. 1 ▶). In order to record more complete data using crystals of lower symmetry, Fourme and coworkers developed a special splinter made of low-absorbing boron nitride in order to mechanically force the crystals to take different orientations within the DAC (Girard *et al.*, 2007 ▶). Since the space group of the SoIPMDH–IPM crystals is *C*2, we placed three crystals into the pressure cell at one time in order to collect high-completeness data sets at a given pressure. The different crystals were tiled with different orientations from each other, with the knotted fibres acting as splinters.

To make HPPX measurements possible at AR-NW12A, the beamline diffractometer, including the goniometer head and the direct-beam stopper, were modified to accept the DAC. Data were collected using a wavelength of 0.700 Å, an exposure time of 5–20 s per image, an oscillation angle of 1° and a beam size of 0.1–0.2 mm. Table 1 ▶ summarizes the parameters for data acquisition at each given pressure.

### 2.4. Data processing and structure refinement

The oscillation photographs were processed and scaled using the *HKL*-2000 suite (Otwinowski & Minor, 1997 ▶). During the integration process, the increase in crystal mosaicity was used to check for radiation damage and only frames without serious radiation damage were merged and used for structure analysis. The initial crystal structure of the SoIPMDH–IPM complex at atmospheric pressure was solved with the program *MOLREP* (Vagin & Teplyakov, 2010 ▶) from the *CCP*4 suite (Winn *et al.*, 2011 ▶), using a monomer of IPMDH from *Thiobacillus ferrooxidans* (PDB entry 1a05; Imada *et al.*, 1998 ▶) as the search model. The structure of the SoIPMDH–IPM complex was then refined with the program *REFMAC*5 (Murshudov *et al.*, 2011 ▶) and manually fitted with *Coot* (Emsley & Cowtan, 2004 ▶). Each of the crystal structure of the SoIPMDH–IPM complex at a series of high pressures was manually refined by iterative cycles between *REFMAC*5 and model building in *Coot*, using the structure of the SoIPMDH–IPM complex at atmospheric pressure as the starting model. Atomic coordinates and structure factors of SoIPMDH–IPM at a series of pressures have been deposited in the Protein Data Bank with codes 3vkz, 3vl2, 3vl3, 3vl4, 3vl6 and 3vl7 for 0.1, 160, 340, 410, 580 and 650 MPa, respectively.

Figures were drawn with the *PyMOL* visualization software (DeLano, 2002 ▶). The vectors in Fig. 5 were made using the modevector.py script from the PyMOL Wiki website (http://www.pymolwiki.org). The internal cavities in Figs. 6(*a*) and 6(*b*) were calculated with *HOLLOW* (Ho & Gruswitz, 2008 ▶). The volumes of the individual molecules without bound waters, IPM or magnesium cations were calculated with *VOIDOO* using a probe radius of 1.4 Å (Kleywegt & Jones, 1994 ▶). The volumes of the internal cavity were calculated with *CASTp* using a probe radius of 1.4 Å (Dundas *et al.*, 2006 ▶). The root-mean-square deviations between equivalent C^α^ atoms in the structures at high pressure and atmospheric pressure were calculated with *SSM* (Schneider, 2002 ▶).

## 3. Results

### 3.1. Compressibility of the unit cell and the SoIPMDH dimer

**Figure 2:**
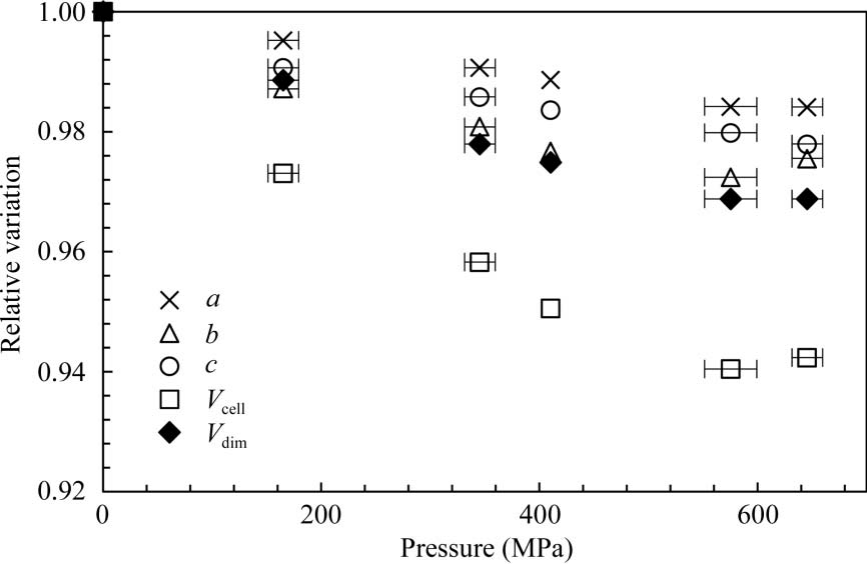
Relative variations of the unit-cell parameters *a*/*a*_o_ (crosses), *b*/*b*_o_ (open triangles), *c*/*c*_o_ (open circles) and the unit-cell volume *V*_cell_/*V*_cell,o_ (open squares), together with the relative variation of the SoIPMDH dimer volume *V*_dim_/*V*_dim,o_ (closed diamonds), as a function of pressure. The errors in the relative variations of the unit-cell parameters were smaller than the symbol sizes.

2 Å resolution crystal structures of the SoIPMDH–IPM complex were determined at room temperature at pressures of 0.1, 160, 340, 410, 580 and 650 MPa. The crystals diffracted reasonably well at pressures as high as 650 MPa, but lost their diffraction entirely at 740 MPa. The compressibilities β were estimated as a linear approximation using
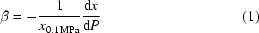
from the relative variation plot of Fig. 2 ▶, where *x* is the unit-cell parameters *a*, *b*, *c*, the unit-cell volume *V*_cell_ or the dimer volume *V*_dim_. The estimated compressibilities of the unit-cell parameters *a*, *b*, *c* and the unit-cell volume are 2.6 × 10^−2^, 4.7 × 10^−2^, 3.6 × 10^−2^ and 0.10 GPa^−1^, respectively. The estimated compressibility for the total volume of the SoIPMDH dimer is 5.4 × 10^−2^ GPa^−1^, which is comparable to that of other proteins previously studied by HPPX. For example, the compressibility of hen egg-white lysozyme was 4.7 × 10^−2^ GPa^−1^ (Kundrot & Richards, 1987 ▶) and that of bovine erythrocyte Cu,Zn superoxide dismutase was 4.0 × 10^ 2^ GPa^−1^ (Ascone *et al.*, 2010 ▶).

### 3.2. Displacement between the structures at different pressures

**Figure 3:**
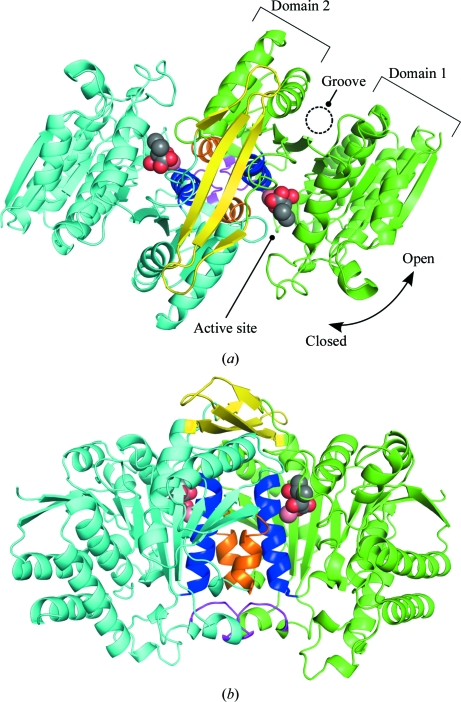
Overall structure of the SoIPMDH–IPM complex. (*a*) Cartoon representation of the overall structure of the SoIPMDH dimer at 0.1 MPa (green, subunit *A*; cyan, subunit *B*; blue, helix *g*; orange, helix *h*; magenta, *FG* loop; yellow, arm-like region). (*b*) Image rotated by 90° compared with (*a*). The IPM substrate molecules and the calcium cations located in the active site of each subunit are represented as spheres.

The SoIPMDH monomer is composed of two domains, referred to as domain 1 and domain 2, connected by a hinge region (Fig. 3 ▶ *a*). The relative orientation of the two domains defines whether the enzyme is in the open form or in the closed form. In this study, the SoIPMDH dimer was crystallized in complex with its substrate, the IPM molecule. As a consequence, the SoIPMDH dimer adopts a closed conformation through binding IPM. An overview of the SoIPMDH dimer at 0.1 MPa is shown in Fig. 3 ▶. Characteristic interactions between the two subunits of the SoIPMDH dimer could be identified, which consist of intersubunit hydrophobic inter­actions between helices *g* and *h*, hydrophobic interactions between the *FG* loops and some hydrophilic inter­actions between the arm-like regions of each subunit, accompanied by the formation of an intersubunit β-­sheet.

**Figure 4:**
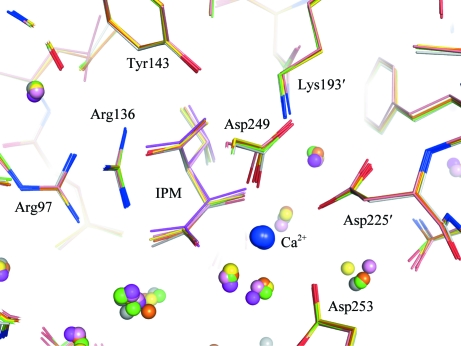
Similarities of the structures at atmospheric and high pressure around the active site of SoIPMDH. The superimposed structures are coloured as follows: white, 0.1 MPa; green, 160 MPa; yellow, 340 MPa; orange, 410 MPa; pink, 580 MPa; magenta, 650 MPa. Water molecules are shown as small spheres.

**Figure 5:**
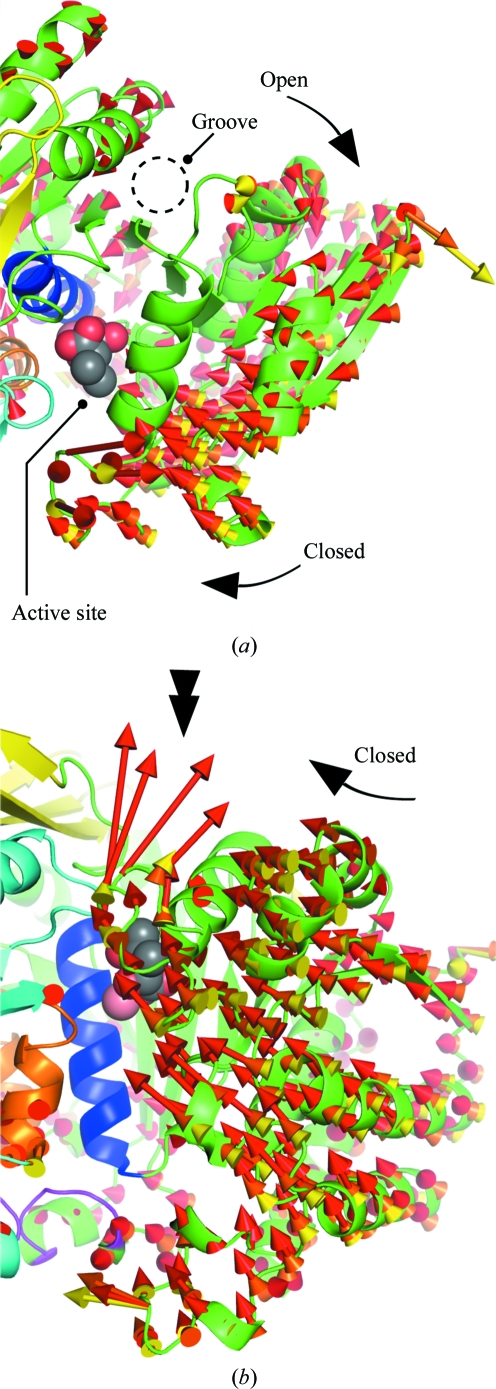
General displacement between structures at a series of pressures. The vectors between the C^α^ positions of the atmospheric and the high-pressure structures are represented as arrows (yellow, 340 MPa–0.1 MPa; orange, 410 MPa–0.1 MPa; red, 580 MPa–0.1 MPa). For an easier visualization, the lengths of the vectors are magnified eight times. (*a*) and (*b*) are enlarged views of Figs. 3 ▶(*a*) and 3 ▶(*b*), respectively. Simultaneous opening of the groove and closure of the active-site entrance can be observed. The double arrow in (*b*) emphasizes the vectors that represent the large motion described by the Lys81–Glu86 loop.

In the structures at 0.1–650 MPa, with the exception of flexible loops on the surface of SoIPMDH and residues that adopt indistinct multiple conformations, no characteristic structural changes were observed. As emphasized by superimposition of the structures around the active site (Fig. 4 ▶), the active-site residues Tyr143, Lys193′ and Asp225 and residues involved in the binding of IPM, *i.e.* Arg97 and Arg136, stay in a fixed position with only small deviations. As a consequence, local structure changes such as side-chain flipping of active-site residues induced by pressure are unlikely to cause a reduction in the activity of SoIPMDH. Nevertheless, global changes induced by pressure can be observed. The mean r.m.s. deviations among the C^α^-atom positions of the 0.1 MPa and the 160, 340, 410, 580 and 650 MPa structures were estimated to be 0.23, 0.33, 0.37, 0.52 and 0.56 Å, respectively. Fig. 5 ▶ shows vectors representing displacements from the C^α^-atom positions in the ambient-pressure structure to those in the high-pressure structures, the lengths of which are magnified eightfold for clarity. Fig. 5 ▶(*a*) shows the vectors viewed from the same angle as in Fig. 3 ▶(*a*), in which the dimer is looked upon from the upper side of the entrance of the active site. Similarly, the vectors in Fig. 5 ▶(*b*) are shown in the same orientation as in Fig. 3 ▶(*b*). The vectors for each high pressure tend to face in approximately the same direction and their lengths increase as the pressure increases. The homogeneous direction of the vectors illustrates the closure of the entrance to the active site when the pressure is increased. In Fig. 5 ▶(*a*) we can observe the opening of the groove of the active site, which occurs simultaneously with the closure of the entrance. Large displacements occur between 0.1 and 580 MPa around the top of the entrance (Fig. 5 ▶ *b*, black double arrow). However, this large displacement is dubious because the electron density in this region is not clear and the model of this region is not well defined.

### 3.3. Volume change of internal cavities in the SoIPMDH dimer

**Figure 6:**
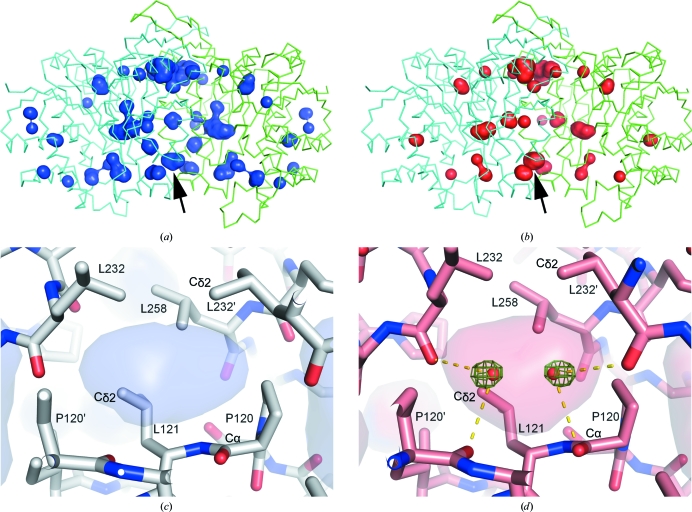
Internal cavities of the SoIPMDH dimer and observed water penetration. (*a*) and (*b*) show internal cavities of the SoIPMDH dimer as surface representations at 0.1 and 580 MPa, respectively. The cavity for which the volume increases with pressure is indicated by an arrow. (*c*) and (*d*) are magnified views around the cavity at 0.1 and 580 MPa, respectively. A difference electron-density map is shown as a green mesh contoured at 4.0σ. No positive peaks are observed in (*c*) at 0.1 MPa. The transparent surfaces in (*c*) and (*d*) represent the cavities; hydrogen bonds are also shown in (*d*).

Most of the cavities are compressed and decrease in volume as the pressure increases (Figs. 6 ▶ *a*, 6 ▶ *b* and 7 ▶). The compressibility of the total volume of the internal cavities is estimated to be 0.73 GPa^−1^. Although most of the cavities are monotonically compressed as pressure increases, the volume of the cavity at the dimer interface (arrow in Figs. 6 ▶ *a* and 6 ▶ *b*) does not decrease with pressure. The volume of this cavity shows an initial decrease in volume, with values of 55.6 and 50.4 Å^3^ at pressures of 0.1 and 160 MPa, respectively, followed by a constant volume increase with values of 54.2, 57.6, 64.7 and 65.5 Å^3^ at 340, 410, 580 and 650 MPa, respectively. This volume change of the cavity is produced by movement of the surrounding residues. For example, the distance between Leu121 C^δ2^ and Leu121′ C^δ2^ changes from 6.6 to 6.5, 7.3, 7.2, 7.7 and 7.4 Å, and the distance between Pro120 C^α^ and Leu232′ C^δ2^ changes from 6.6 to 6.5, 6.4, 6.6, 7.1 and 7.5 Å at pressures of 0.1 to 160, 340, 410, 580 and 650 MPa, respectively. These distances tend to decrease up to moderately high pressures (160 or 340 MPa) and increase at higher pressures in correspondence to the cavity-volume change.

### 3.4. Water penetration into the internal cavity located at the dimer interface

In parallel with the volume increase reported above, the penetration of water molecules in the cavity can be observed, as illustrated by difference electron-density maps around the cavities of the 0.1 and 580 MPa structures, respectively (Figs. 6 ▶ *c* and 6 ▶ *d*). The cavity is surrounded by hydrophobic residues belonging to both subunits (Pro120, Leu121, Ile125, Leu232 and Leu258). No electron density could be observed in the cavity at 0.1 MPa, while at 580 MPa two peaks were clearly visible. It is noteworthy that these positive densities started to appear at 410 MPa (data not shown). By taking the buffer composition and the hydrogen-bond distances separating both peaks from the backbone carbonyl O atoms of the closest Pro120 and Leu232 into account, these densities are assumed to belong to water molecules. The distances between the peak and the carbonyl O atom of Pro120 are 3.0, 3.1 and 3.0 Å, and those for Leu232 are 3.6, 3.3 and 3.4 Å at 410, 580 and 650 MPa, respectively. The distance between the two peaks is short (2.8, 2.4 and 2.5 Å at 410, 580 and 650 MPa, respectively) and the two positions are crystallographically equivalent and related by the twofold axis.

A quick estimation of the occupancy of the water molecules was performed by varying their occupancy from 0.3 to 0.8 through iterative refinement in order to adjust their *B* factor to that of the nearest carbonyl O atom (Pro120), corresponding to 11.2, 7.3 and 13.0 Å^2^ at 410, 580 and 650 MPa, respectively. The estimated occupancy of the penetrating water was 0.42 at 410 MPa and increased to 0.51 and 0.76 at 580 and 650 MPa, respectively. This increase in the water-molecule occupancy emphasizes the intensification of water penetration into the cavity as the pressure increases.

### 3.5. Generation of an additional cleft accompanied by penetration of water molecules

The opposite side of the SoIPMDH active site forms a groove along the central β-sheet, with Pro108 and Leu305 located at the bottom (Fig. 8 ▶ *a*). The separation between the side chains of Pro108 and Leu305 increases with pressure; notably, the distance between Pro108 C^δ^ and Leu305 C^δ1^ varies from 4.8 to 5.0, 5.1, 5.6 and 5.9 Å at pressures of 0.1 to 340, 410, 580 and 650 MPa, respectively. Similarly, the distance between Pro10 C^γ^ and Leu305 C^δ2^ increases from 5.0 to 5.1, 5.4, 5.5 and 5.8 Å at 0.1 to 340, 410, 580 and 650 MPa, respectively. The structural change from 0.1 to 580 MPa is characterized by the appearance of three peaks in the difference electron-density map between Pro108 and Leu305 (water molecules W697, W698 and W699; Figs. 8 ▶ *b* and 8 ▶ *c*). At 580 MPa a new cleft that was not observed in the 0.1 MPa structure is formed between Pro108 and Leu305 as visualized on a solvent-excluded surface drawn using a probe radius of 1.4 Å (Fig. 8 ▶ *d*). This newly generated crevice is large enough to accommodate the two penetrating water molecules W697 and W698. This conformational change illustrates a deeper penetration of water molecules into the protein at 580 MPa. The additional water molecules are stabilized by hydrogen bonds (Fig. 8 ▶ *e*), with distances of 3.2, 2.6 and 3.0 Å for W697⋯Ser266 O^γ^, W697⋯W698 and W697⋯W625, of 2.5 and 2.9 Å for W698⋯W699 and W698⋯Leu106 carbonyl O atom, of 3.2 Å for W699⋯His309 N^∊2^ and of 2.8 and 2.9 Å for W625⋯Pro108 carbonyl O atom and W625⋯Ser266 amide N atom, respectively.

## 4. Discussion

### 4.1. Water penetration into the hydrophobic cavity and destabilization of the SoIPMDH dimer

**Figure 7:**
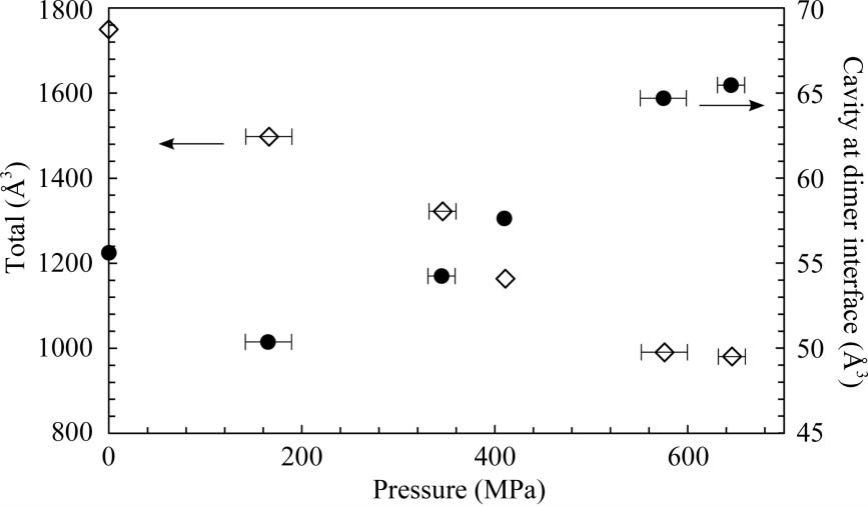
Variations in the total volume of internal cavities (open diamonds) of the SoIPMDH dimer and the volume of the cavity at the dimer interface (closed circles) as a function of pressure.

**Figure 8:**
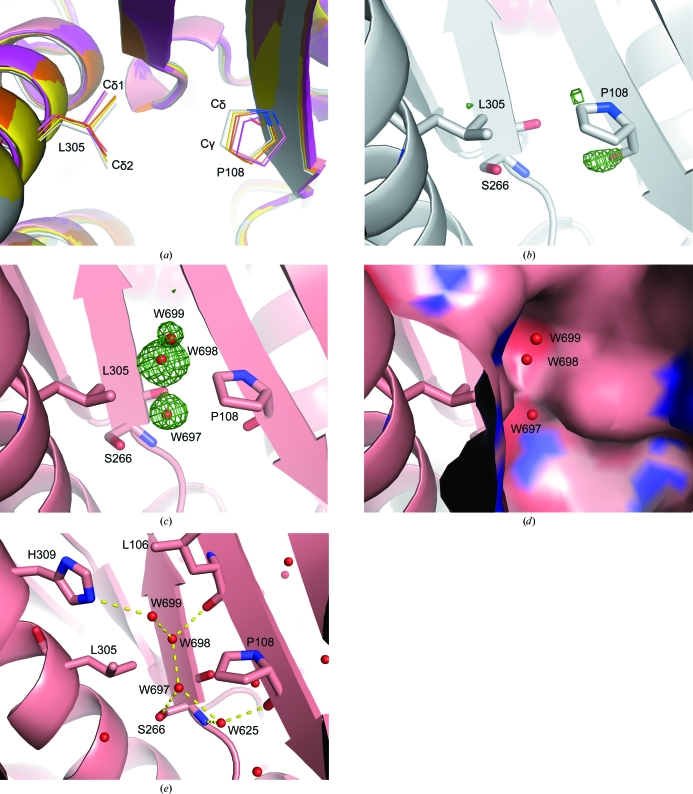
Generation of a new cleft at the bottom of the groove and water penetration into the cleft at 580 MPa. (*a*) Pro108 and Leu305 at the bottom of the groove on the opposite side to the active site at a series of pressures. The main chains are shown as cartoon representations (white, 0.1 MPa; yellow, 340 MPa; orange, 410 MPa; pink, 580 MPa; magenta, 650 MPa). Pro108 and Leu305 are represented as wires (red, O atoms; blue, N atoms). (*b*, *c*) Difference electron-density map between Pro108 and Leu305 shown as a green mesh contoured at 3.0σ at 0.1 and 580 MPa, respectively. Pro108, Ser266 and Leu305 are represented as sticks. Three positive peaks are observed at 580 MPa in (*c*) and were assigned as three water molecules: W697, W698 and W699 (represented by red balls). No positive peaks are observed in the cleft at 0.1 MPa in (*b*). (*d*) Solvent-excluded surface representation around Pro108 and Leu305 at 580 MPa, drawn using a probe radius of 1.4 Å. (*e*) Hydrogen-bond network of the penetrating water molecules W697, W698 and W699 as illustrated by yellow dashed lines

Under high-pressure stress, proteins adopt a state of lower partial molar volume (PMV) according to Le Chatelier’s principle. The PMV of a macromolecule is expressed as the summation of the van der Waals volume, the void volume and the volume change caused by hydration. At 160 MPa, the system reduces the PMV of the SoIPMDH dimer by compressing the interior cavities (including the cavity at the dimer interface). At pressures over 410 MPa, the system reduces the PMV of the dimer by filling the cavity at the dimer interface with water molecules from the bulk region, in addition to compressing the other cavities within the interior of the protein, which results in an overall reduction of the PMV. The occupancy of the penetrating water molecules increases with pressure, which implies a rise in the rate of penetration of the water molecules into the cavity, partly explaining the swelling of the cavity volume with pressure. The water filling of the cavity probably plays a role by cancelling the void volume of the cavity, despite apparently increasing the total volume of the cavity. As shown in Fig. 7 ▶, the volume of the cavity starts to increase above 340 MPa, which might indicate the beginning of the entrance of the water molecules into the cavity. At 650 MPa, both water molecules in the cavity at the dimer interface show nearly full occupancies, which might initiate the destabilization of the dimer itself because of the repulsion generated between the too closely positioned water molecules. In previously published HPPX and MD simulation work on L99A-mutated T4 lysozyme, water penetration into the large hydrophobic cavity (∼160 Å^3^) created by the mutation was observed at high pressure (Collins *et al.*, 2005 ▶). These results illustrated that at least two, and possibly as many as four, water molecules simultaneously penetrated into the large cavity at high pressure, while the volume of the cavity decreased by 3%. In the case of T4 lysozyme, there is no need to increase the volume of the cavity prior to accommodating water molecules because its volume is already large enough. In the case of SoIPMDH, the volume of the cavity at the dimer interface is only 56 Å^3^ at 0.1 MPa and reduces slightly to 50 Å^3^ at 160 MPa. As a consequence, there is a need to increase the volume of the cavity at higher pressures (over 340 MPa) in order to accommodate additional water molecules.

### 4.2. Generation of the cleft accompanied by water penetration

In the 580 and 650 MPa structures, an additional cleft is generated at the bottom of the groove on the opposite side to the active site. Two water molecules W697 and W698 appeared inside the cleft, together with another water molecule W699 near the groove. These three waters were not observed in the structures at lower pressures ranging from 0.1 to 410 MPa. The generation of the cleft together with water penetration can be considered as an initial step in the pressure-denaturation process of SoIPMDH. In the 580 MPa structure, Ser266 O^γ^ forms a hydrogen bond to W697, allowing anchoring of W697 into the cleft. Additionally, the three new water molecules form a hydrogen-bond network among each other as well as with the atoms of the protein. In the structures at pressures ranging from 0.1 to 410 MPa, the solvent-excluded surface at the bottom of the groove is nonpolar since it is mostly composed of the Pro108 and Leu305 side chains. As a consequence, the penetrating water molecules, especially W697 and W698, force this nonpolar surface to open. In the previously published work of Imai and coworkers, the change in PMV associated with the pressure-induced conformation change of ubiquitin was analyzed using a three-dimensional RISM theory of molecular solvation (Imai *et al.*, 2007 ▶). Their study showed that the conformational change from a closed to an open form of ubiquitin at high pressure is the main cause of the reduction in PMV, which can be ascribed to water penetration into the hydrophobic core of the protein. The authors subsequently performed a 1 µs high-pressure MD simulation of ubiquitin and observed the conformational change accompanied by water penetration (Imai & Sugita, 2010 ▶). The present structural aspect of the SoIPMDH cleft with water penetration strongly resembles that of the computational study of ubiquitin denaturation. In the case of ubiquitin, a water molecule penetrates deeply into the cleft when the protein is in its open state and is buried among internal hydrophobic side chains. Similarly, in the case of SoIPMDH water molecules also penetrate the cleft and are buried among the internal hydrophobic side chains of Leu106, Pro108, Ala268 and Leu305. The SoIPMDH surface at the cleft is not completely hydrophobic, but rather shows some hydrophilic properties, notably owing to the Leu106 carbonyl O atom, the Ser266 amide N and O^γ^ atoms and His309 N^∊2^. The hydrogen-bond network resulting from these polar groups contributes to the proper localization of the water molecules. Thus, the generation of the cleft accompanied by water penetration observed in our study may be a representation of the conformational change driven by the reduction of the PMV induced by the pressure increase.

More precisely, the hydrogen bond between W697 and Ser266 O^γ^ seems to play an important role in this structural change. SoIPMDH from the nonpiezophile *S. oneidensis* MR-1 contains a serine at position 266, while IPMDH from the obligate piezophile *S. benthica* DB21MT-2 (SbIPMDH) contains an alanine at the same position. A possible explan­ation of the different piezo-sensitivity of SbIPMDH could be that a water molecule cannot be stabilized at the same position as W697 because of the lack of the side chain in Ala266, which would cause the formation of a limited hydrogen-bond network. We therefore propose that the mutation of Ser266 in SoIPMDH to alanine may be characterized by a different behaviour of the protein at 580 MPa. On the other hand, the amino-acid residues surrounding the internal hydrophobic cavity (Fig. 6 ▶) are conserved in SoIPMDH and SbIPMDH. Thus, the water penetration into this cavity does not seem to be related to the difference in the pressure tolerances of SoIPMDH and SbIPMDH.

As shown in Table 1 ▶, the number of water molecules found at the SoIPMDH surface increases up to 410 MPa. High-pressure simulations showed an augmentation of the inter­actions of proteins with water (Marchi & Akasaka, 2001 ▶). Additionally, an increase in the number of ordered water molecules with pressure has been reported for HPPX using lysozyme (Kundrot & Richards, 1987 ▶) and cubic cowpea mosaic virus (Girard *et al.*, 2005 ▶), in which an increase in the number of hydrogen bonds between the water molecules and the protein was observed. In the case of SoIPMDH, the number of waters decreases again above 580 MPa. We think that this might be caused by the start of pressure denaturation as we have found water penetration into the cavity and the cleft, but systematic changes in the water network that would correspond to a reduction of the number of structured waters found were not clearly observed.

## 5. Conclusion

Structures of SoIPMDH at pressures ranging from 0.1 to 650 MPa were determined at about 2 Å resolution using the HPPX method. A thorough comparison of these structures gives a detailed picture of the phenomena induced by high pressure on the protein, such as compression of the protein cavities, water penetration into hydrophobic cavities and the generation of a new cleft on the molecular surface accompanied by water penetration. These observations qualitatively agree with the computational and experimental results reported previously (Imai *et al.*, 2007 ▶; Imai & Sugita, 2010 ▶; Harano & Kinoshita, 2006 ▶). The water penetration into the hydrophobic regions of SoIPMDH is supported by hydrogen bonding to polar regions which are interspersed on the hydrophobic surface of the protein, such as the carbonyl O atom of Pro120 or Ser266 O^γ^. The role of the penetrating water molecules in reducing the PMV will be confirmed by estimating how much the PMV is reduced by water penetration into the cavity or the cleft using computational methods such as the three-dimensional RISM theory (Imai *et al.*, 2007 ▶) or the three-dimensional integral equation theory (Harano & Kinoshita, 2006 ▶). In addition, the comparison of high-pressure structures of SoIPMDH with those of IPMDH from deep-sea bacteria will illuminate the mechanism of pressure tolerance and will deepen our understanding of how piezophiles adapt their enzymes to extreme high-pressure environments in deep seas.

## Supplementary Material

PDB reference: SoIPMDH–IPM, 0.1 MPa, 3vkz

PDB reference: 160 MPa, 3vl2

PDB reference: 340 MPa, 3vl3

PDB reference: 410 MPa, 3vl4

PDB reference: 580 MPa, 3vl6

PDB reference: 650 MPa, 3vl7

## Figures and Tables

**Table 1:** Data-collection parameters and refinement statistics of SoIPMDH–IPM crystals at a series of pressures Values in parentheses are for the highest resolution shell.

Pressure (MPa)	0.1	160	340	410	580	650
Data collection						
Beamline or X-ray generator	FR-E SuperBright	PF-AR NW12A				
Detector	R-AXIS VII	ADSC Quantum 210r				
Temperature	Room temperature					
Wavelength (Å)	1.5418	0.700				
Oscillation angle per frame (°)	1					
Exposure time per frame (s)	30	5	10	5	10–20	5
Crystal-to-detector distance (mm)	95	300	200	250	250	250
No. of crystals used	3	2	3	3	3	3
Space group	*C*2					
Unit-cell parameters						
*a* (Å)	104.797 (5)	104.298 (2)	103.824 (2)	103.610 (5)	103.144 (2)	103.135 (5)
*b* (Å)	59.003 (2)	58.251 (2)	57.877 (1)	57.633 (3)	57.380 (2)	57.561 (5)
*c* (Å)	76.754 (6)	76.044 (3)	75.671 (2)	75.504 (4)	75.213 (2)	75.067 (2)
β (°)	119.070 (2)	119.090 (2)	119.023 (1)	118.978 (4)	118.758 (1)	118.668 (3)
Resolution range (Å)	50.00–1.84 (1.87–1.84)	50.00–2.06 (2.10–2.06)	50.00–1.80 (1.83–1.80)	50.00–1.88 (1.92–1.88)	50.00–2.07 (2.11–2.07)	50.00–2.20 (2.24–2.20)
No. of reflections	31951 (1612)	20579 (1019)	33575 (1697)	27609 (1469)	22194 (1152)	17498 (882)
*R*_merge_† (%)	6.2 (39.9)	4.9 (15.7)	5.3 (31.5)	7.2 (39.8)	8.5 (37.7)	9.9 (35.4)
Completeness (%)	89.6 (89.3)	83.2 (85.9)	91.6 (95.6)	86.4 (91.5)	94.0 (95.0)	88.9 (91.9)
〈*I*/σ(*I*)〉	22.1	14.5	15.7	10.5	9.8	10.2
Multiplicity	3.8 (3.8)	2.2 (1.9)	2.9 (2.8)	2.8 (2.7)	4.2 (4.2)	2.7 (2.7)
Refinement						
*R*_work_‡ (%)	14.20	13.54	15.70	15.60	17.35	16.56
*R*_free_§ (%)	18.49	19.21	20.20	21.29	23.46	24.42
No. of atoms						
Protein	2807	2807	2807	2807	2807	2807
Ligand/ion	14	14	14	14	14	14
Water	184	204	275	273	199	176
*B* factor (Å^2^)						
Protein	25.62	18.75	18.20	17.62	16.55	16.86
Ligand/ion	18.08	12.26	12.42	12.68	14.02	17.20
Water	36.32	28.37	30.23	29.00	22.94	20.99
R.m.s.d. from ideality						
Bond lengths (Å)	0.029	0.024	0.030	0.024	0.023	0.018
Bond angles (°)	2.207	1.941	2.401	2.008	1.966	1.761

† *R*_merge_ is defined as 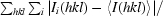 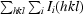, where *I_i_*(*hkl*) is the *i*th observation of reflection *hkl* and 〈*I*(*hkl*)〉 is the weighted mean of all observations (after rejection of outliers).

‡ *R*_work_ is defined as 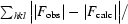 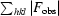.

§ *R*_free_ is calculated using 5% of the data that were randomly chosen and excluded from the refinement.
